# Supplementary material for: Reduced metabolism supports hypoxic flight in the high-flying bar-headed goose (Anser indicus)
Source: eLife. 2019 Sep 3;8:e44986. doi: 10.7554/eLife.44986 (PMC6721836; doi:10.7554/eLife.44986)
Supplement: Supplementary file 1. — Values are mean ± SEM. Asterisks indicate significant difference from normoxia (ANOVA; * indicates p<0.05; ** indicates p<0.01; *** indicates p<0.001). [file elife-44986-supp1.docx]

Supplementary file 1

|  | **Normoxia**  **0.21 F_i_O_2_** | **Moderate hypoxia**  **0.105 F_i_O_2_** | **Severe hypoxia**  **0.07 F_i_O_2_** |
| --- | --- | --- | --- |
| **Flight length (sec)**  **Mean ± se (range)** | 300 ± 37  (97-663) | 197 ± 19  (84-461) | 140 ± 35**  (60-468) |
| **n Birds** | 1 | 1 | 1 |
| **n Flights** | 20 | 25 | 11 |
| **Rest:** |  |  |  |
| ${\dot{\boldsymbol{V}}}_{\boldsymbol{O}\mathbf{2}}$**(ml O_2_ min^-1^ kg^-1^)** | 10.5 ± 0.6 | (-) | (-) |
| ${\dot{\boldsymbol{V}}}_{\boldsymbol{CO}\mathbf{2}}$**(ml CO_2_ min^-1^ kg^-1^)** | 7.67 ± 0.48 | 6.24 ± 0.41* | 6.47 ± 0.49 |
| **RER** | 0.73 ± 0.02 | (-) | (-) |
| **Heart rate (bpm)** | 104.1 ± 3.2 | 103.5 ± 3.9 | 117.3 ± 3.9 |
| **CO_2_ pulse (ml CO_2_ beat^-1^ kg^-1^)** | 0.075 ± 0.005 | 0.060 ± 0.004 | 0.059 ± 0.008 |
| **Arterial** $\mathbf{P}\mathbf{o}_{\mathbf{2}}$ **(mmHg)** | 53.2 ± 30 | 21.6 ± 10.6 | 13.0 ± 5.5 |
| **Venous** $\mathbf{P}\mathbf{o}_{\mathbf{2}}$ **(mmHg)** | 36.1 ± 8.3 | 20.0 ± 11.6 | 20.9 ± 0.45 |
| **Venous temperature (˚C)** | 40.6 | 40.4 | 40.6 |
| **Pre-flight:** |  |  |  |
| ${\dot{\boldsymbol{V}}}_{\boldsymbol{O}\mathbf{2}}$**(ml O_2_ min^-1^ kg^-1^)** | 12.7 ± 0.8 | (-) | (-) |
| ${\dot{\boldsymbol{V}}}_{\boldsymbol{CO}\mathbf{2}}$**(ml CO_2_ min^-1^ kg^-1^)** | 10.1 ± 0.9 | 6.01 ± 0.6** | 8.38 ± 1.4 |
| **RER** | 0.79 ± 0.03 | (-) | (-) |
| **Heart rate (bpm)** | 124.9 ± 15 | 129.9 ± 8.3 | 149.9 ± 11 |
| **CO_2_ pulse (ml CO_2_ beat^-1^ kg^-1^)** | 0.065 ± 0.007 | 0.051 ± 0.005 | 0.057 ± 0.009 |
| **Arterial** $\mathbf{P}\mathbf{o}_{\mathbf{2}}$ **(mmHg)** | 72.1 ± 0.42 | 56.5 ± 5.4* | 36.7 ± 0.54*** |
| **Venous** $\mathbf{P}\mathbf{o}_{\mathbf{2}}$ **(mmHg)** | 40.0 ± 1.0 | 42.0 ± 1.7 | 35.2 ± 3.2 |
| **Venous temperature (˚C)** | 40.6 ± 0.14 | 40.7 ± 0.26 | 40.4 ± 0.37 |
| **Flight:** |  |  |  |
| ${\dot{\boldsymbol{V}}}_{\boldsymbol{O}\mathbf{2}}$**(ml O_2_ min^-1^ kg^-1^)** | 180.4 ± 7.4 | (-) | (-) |
| ${\dot{\boldsymbol{V}}}_{\boldsymbol{CO}\mathbf{2}}$**(ml CO_2_ min^-1^ kg^-1^)** | 157.4 ± 8.4 | 162.9 ± 6.5 | 126.6 ± 4.6* |
| **RER** | 0.87 ± 0.01 | (-) | (-) |
| **Heart rate (bpm)** | 333.6 ± 11 | 322.6 ± 12 | 293.7 ± 18 |
| **CO_2_ pulse (ml CO_2_ beat^-1^ kg^-1^)** | 0.45 ± 0.03 | 0.51 ± 0.02 | 0.45 ± 0.04 |
| **Arterial** $\mathbf{P}\mathbf{o}_{\mathbf{2}}$ **(mmHg)** | 85.8 ± 10.8 | 47.0 ± 4.3** | 36.2 ± 2.7** |
| **Venous** $\mathbf{P}\mathbf{o}_{\mathbf{2}}$ **(mmHg)** | 42.8 ± 1.5 | 30.3 ± 0.73*** | 26.2 ± 0.37*** |
| **Venous temperature (˚C)** | 39.4 ± 0.28 | 39.3 ± 0.27 | 39.0 ± 0.21 |
