# Supplementary material for: Reduced metabolism supports hypoxic flight in the high-flying bar-headed goose (Anser indicus)
Source: eLife. 2019 Sep 3;8:e44986. doi: 10.7554/eLife.44986 (PMC6721836; doi:10.7554/eLife.44986)
Supplement: Supplementary file 2. — Values are mean ± SEM. Asterisks indicate significant difference from normoxia (linear mixed model ANOVA; * indicates p<0.05; ** indicates p<0.01; *** indicates p<0.001). [file elife-44986-supp2.docx]

Supplementary file 2

| **Mixed venous** | **Normoxia**  **0.21 F_i_O_2_** | **Moderate hypoxia**  **0.105 F_i_O_2_** | **Severe hypoxia**  **0.07 F_i_O_2_** |
| --- | --- | --- | --- |
| **Flight length (sec)**  **Mean ± se (range)** | 125 ± 18  (59-237) | 127 ± 32  (30-260) | 97 ± 28  (67-193) |
| **n Birds** | 4 | 2 | 1 |
| **n Flights** | 13 | 6 | 3 |
| **Rest:** |  |  |  |
| **Venous** $\mathbf{P}\mathbf{o}_{\mathbf{2}}$ **(mmHg)** | 46.5 ± 2.9 | 28.9 ± 3.0*** | 23.1 ± 1.8*** |
| **Venous temperature (˚C)** | 40.8 ± 0.14 | 40.7 ± 0.24 | 40.9 ± 0.20 |
| **Pre-flight:** |  |  |  |
| ${\dot{\boldsymbol{V}}}_{\boldsymbol{O}\mathbf{2}}$**(ml O_2_ min^-1^ kg^-1^)** | 17.0 ± 1.5 | (-) | (-) |
| ${\dot{\boldsymbol{V}}}_{\boldsymbol{CO}\mathbf{2}}$**(ml CO_2_ min^-1^ kg^-1^)** | 11.6 ± 1.0 | 8.07 ± 1.9 | 10.6 ± 1 |
| **RER** | 0.70 ± 0.03 | (-) | (-) |
| **Heart rate (bpm)** | 150 ± 7.5 | 164.1 ± 9.5 | 146.5 ± 17.5 |
| **Venous** $\mathbf{P}\mathbf{o}_{\mathbf{2}}$ **(mmHg)** | 47.07 ± 2.9 | 42.3 ± 1.2 | 35.2 ± 3.2 |
| **Venous temperature (˚C)** | 41.1 ± 0.15 | 40.7 ± 0.26 | 40.4 ± 0.37 |
| **Flight (start):** |  |  |  |
| ${\dot{\boldsymbol{V}}}_{\boldsymbol{O}\mathbf{2}}$**(ml O_2_ min^-1^ kg^-1^)** | 223.1 ± 7.7 | (-) | (-) |
| ${\dot{\boldsymbol{V}}}_{\boldsymbol{CO}\mathbf{2}}$**(ml CO_2_ min^-1^ kg^-1^)** | 224.1 ± 15.1 | 187.9 ± 16.4 | 138.7 ± 15.4* |
| **RER** | 0.99 ± 0.04 | (-) | (-) |
| **Heart rate (bpm)** | 260.3 ± 12.5 | 257.1 ± 21.9 | 273.4 ± 3.8 |
| **Venous** $\mathbf{P}\mathbf{o}_{\mathbf{2}}$ **(mmHg)** | 49.4 ± 2.9 | 32.5 ± 4.4** | 30.2 ± 0.1** |
| **Venous temperature (˚C)** | 40.9 ± 0.18 | 40.6 ± 0.24 | 40.4 ± 0.30 |
| **Flight (steady state):** |  |  |  |
| ${\dot{\boldsymbol{V}}}_{\boldsymbol{O}\mathbf{2}}$**(ml O_2_ min^-1^ kg^-1^)** | 225.8 ± 9.3 | (-) | (-) |
| ${\dot{\boldsymbol{V}}}_{\boldsymbol{CO}\mathbf{2}}$**(ml CO_2_ min^-1^ kg^-1^)** | 221.2 ± 15.4 | 169.3 ± 16.6* | 122.1 ± 0.8** |
| **RER** | 0.97 ± 0.03 | (-) | (-) |
| **Heart rate (bpm)** | 298.8 ± 12.0 | 304.1 ± 18.7 | 311.8 ± 24.4 |
| **Venous** $\mathbf{P}\mathbf{o}_{\mathbf{2}}$ **(mmHg)** | 41.8 ± 1.1 | 30.9 ± 0.82 | 26.2 ± 0.37* |
| **Venous temperature (˚C)** | 39.5 ± 0.38 | 39.3 ± 0.27 | 39.0 ± 0.21 |
| **Flight (end):** |  |  |  |
| ${\dot{\boldsymbol{V}}}_{\boldsymbol{O}\mathbf{2}}$**(ml O_2_ min^-1^ kg^-1^)** | 239.0 ± 5.5 | (-) | (-) |
| ${\dot{\boldsymbol{V}}}_{\boldsymbol{CO}\mathbf{2}}$**(ml CO_2_ min^-1^ kg^-1^)** | 236.6 ± 11.8 | 175.2 ± 16.3** | 128.1 ± 2.2** |
| **RER** | 0.98 ± 0.03 | (-) | (-) |
| **Heart rate (bpm)** | 279.2 ± 6.9 | 290.1 ± 21.7 | 293.6 ± 2.9 |
| **Venous** $\mathbf{P}\mathbf{o}_{\mathbf{2}}$ **(mmHg)** | 41.2 ± 1.3 | 30.9 ± 0.8 | 25.8 ± 0.5* |
| **Venous temperature (˚C)** | 40.1 ± 0.53 | 39.7 ± 0.32 | 38.9 ± 0.12 |
| **Recovery:** |  |  |  |
| ${\dot{\boldsymbol{V}}}_{\boldsymbol{O}\mathbf{2}}$**(ml O_2_ min^-1^ kg^-1^)** | 51.2 ± 10 | (-) | (-) |
| ${\dot{\boldsymbol{V}}}_{\boldsymbol{CO}\mathbf{2}}$**(ml CO_2_ min^-1^ kg^-1^)** | 51.9 ± 10 | 27.1 ± 7.2 | 32.9 ± 3.9 |
| **RER** | 1.00 ± 0.09 | (-) | (-) |
| **Heart rate (bpm)** | 189.4 ± 10.2 | 173.0 ± 11.2 | 191.8 ± 3.5 |
| **Venous** $\mathbf{P}\mathbf{o}_{\mathbf{2}}$ **(mmHg)** | 50.2 ± 3.2 | 36.0 ± 2.4** | 31.4 ± 2.3** |
| **Venous temperature (˚C)** | 40.8 ± 0.19 | 40.1 ± 0.10 | 39.7 ± 0.6 |
