# Supplementary material for: Reduced metabolism supports hypoxic flight in the high-flying bar-headed goose (Anser indicus)
Source: eLife. 2019 Sep 3;8:e44986. doi: 10.7554/eLife.44986 (PMC6721836; doi:10.7554/eLife.44986)
Supplement: Supplementary file 3. — Values are mean ± SEM. Asterisks indicate significant difference from normoxia (ANOVA; * indicates p<0.05; ** indicates p<0.01; *** indicates p<0.001). [file elife-44986-supp3.docx]

Supplementary file 3

| **Arterial** | **Normoxia**  **0.21 F_i_O_2_** | **Moderate hypoxia**  **0.105 F_i_O_2_** | **Severe hypoxia**  **0.07 F_i_O_2_** |
| --- | --- | --- | --- |
| **Flight length (sec)**  **Mean ± se (range)** | 377 ± 33  (344-409) | 215 ± 68  (98-332) | 95.5 ± 15  (60-132) |
| **n Birds** | 1 | 1 | 1 |
| **n Flights** | 2 | 3 | 4 |
| **Rest:** |  |  |  |
| **Arterial** $\mathbf{P}\mathbf{o}_{\mathbf{2}}$ **(mmHg)** | 53.2 ± 30 | 21.6 ± 1.7 | 13.0 ± 5.5 |
| **Pre-flight:** |  |  |  |
| ${\dot{\boldsymbol{V}}}_{\boldsymbol{O}\mathbf{2}}$**(ml O_2_ min^-1^ kg^-1^)** | 12.09 ± 0.7 | (-) | (-) |
| ${\dot{\boldsymbol{V}}}_{\boldsymbol{CO}\mathbf{2}}$**(ml CO_2_ min^-1^ kg^-1^)** | 7.79 ± 0.8 | 5.77 ± 0.9 | 7.64 ± 3.5 |
| **RER** | 0.64 ± 0.03 | (-) | (-) |
| **Heart rate (bpm)** | 160.3 ± 20.5 | 164.5 ± 15.5 | 154.6 ± 10.8 |
| **Arterial** $\mathbf{P}\mathbf{o}_{\mathbf{2}}$ **(mmHg)** | 72.1 ± 0.42 | 56.5 ± 5.4 | 36.7 ± 0.54 |
| **Flight (start):** |  |  |  |
| ${\dot{\boldsymbol{V}}}_{\boldsymbol{O}\mathbf{2}}$**(ml O_2_ min^-1^ kg^-1^)** | 167.1 ± 8.7 | (-) | (-) |
| ${\dot{\boldsymbol{V}}}_{\boldsymbol{CO}\mathbf{2}}$**(ml CO_2_ min^-1^ kg^-1^)** | 141.9 ± 13 | 166.3 ± 10.8 | 137.4 ± 13.1 |
| **RER** | 0.85 ± 0.03 | (-) | (-) |
| **Heart rate (bpm)** | 291.1 ± 2.6 | 277.6 ± 9.1 | 272.3 ± 10.6 |
| **Arterial** $\mathbf{P}\mathbf{o}_{\mathbf{2}}$ **(mmHg)** | 58.6 ± 5.3 | 47.2 ± 1.7 | 34.7 ±1.8** |
| **Flight (steady state):** |  |  |  |
| ${\dot{\boldsymbol{V}}}_{\boldsymbol{O}\mathbf{2}}$**(ml O_2_ min^-1^ kg^-1^)** | 127.7 ± 1.2 | (-) | (-) |
| ${\dot{\boldsymbol{V}}}_{\boldsymbol{CO}\mathbf{2}}$**(ml CO_2_ min^-1^ kg^-1^)** | 105.0 ± 0.8 | 133.3 ± 21 | 126.7 ± 10.4 |
| **RER** | 0.82 ± 0.01 | (-) | (-) |
| **Heart rate (bpm)** | 309.4 ± 3.3 | 209.5 ± 14 | 289.0 ± 30.4 |
| **Arterial** $\mathbf{P}\mathbf{o}_{\mathbf{2}}$ **(mmHg)** | 85.8 ± 10.8 | 47.0 ± 4.3** | 36.2 ± 2.7** |
| **Flight (end):** |  |  |  |
| ${\dot{\boldsymbol{V}}}_{\boldsymbol{O}\mathbf{2}}$**(ml O_2_ min^-1^ kg^-1^)** | 205.0 ± 9.1 | (-) | (-) |
| ${\dot{\boldsymbol{V}}}_{\boldsymbol{CO}\mathbf{2}}$**(ml CO_2_ min^-1^ kg^-1^)** | 183.8 ± 5.5 | 168.5 ± 23 | 140.0 ± 9.1 |
| **RER** | 0.90 ± 0.01 | (-) | (-) |
| **Heart rate (bpm)** | 307.7 ± 17 | 260.4 ± 20 | 288.7 ± 28 |
| **Arterial** $\mathbf{P}\mathbf{o}_{\mathbf{2}}$ **(mmHg)** | 95.7 ± 6.0 | 49.5 ± 2.6*** | 38.2 ± 3.1*** |
| **Recovery:** |  |  |  |
| ${\dot{\boldsymbol{V}}}_{\boldsymbol{O}\mathbf{2}}$**(ml O_2_ min^-1^ kg^-1^)** | 17.2 ± 2.1 | (-) | (-) |
| ${\dot{\boldsymbol{V}}}_{\boldsymbol{CO}\mathbf{2}}$**(ml CO_2_ min^-1^ kg^-1^)** | 12.8 ± 1.2 | 17.5 ± 3.1 | 25.4 ± 4.2 |
| **RER** | 0.76 ± 0.02 | (-) | (-) |
| **Heart rate (bpm)** | 174.4 ± 13.5 | 184.2 ± 24 | 161.3 ± 8.1 |
| **Arterial** $\mathbf{P}\mathbf{o}_{\mathbf{2}}$ **(mmHg)** | 80.1 ± 5.1 | 51.3 ± 12.9 | 46.7 ± 10.3 |
