# Supplementary material for: Reduced metabolism supports hypoxic flight in the high-flying bar-headed goose (Anser indicus)
Source: eLife. 2019 Sep 3;8:e44986. doi: 10.7554/eLife.44986 (PMC6721836; doi:10.7554/eLife.44986)
Supplement: Supplementary file 4. — Data in this table are from a separate unpublished study on the same birds as the primary study. Each mean is the average of 40 wingbeat values. Asterisks (*) indicate statistically significant comparisons between normoxia and moderate hypoxia in a mixed model analysis after posthoc application of the false discovery rate. Frequency is calculated as the inverse of the entire wingbeat duration, not in terms of the upstroke or downstroke alone. [file elife-44986-supp4.docx]

Supplementary file 4

|  |  | Downstroke | | Upstroke | |
| --- | --- | --- | --- | --- | --- |
| Variable description | Variable | Normoxia | Hypoxia | Normoxia | Hypoxia |
| Period (ms) | T | 116.3 ± 3.8 | 114.4 ± 4.0 | 85.6 ± 3.2 | 89.8 ± 3.9 * |
| Wingtip speed (m/s) | U_tip_ | 7.1 ± 0.4 | 7.0 ± 0.4 | 8.0 ± 0.6 | 7.5 ± 0.3 * |
|  | U_x_ | 0.07 ± 0.02 | 0.06 ± 0.03 | 0.10 ± 0.04 | -0.10 ± 0.03 |
| Body speed (m/s) | U_y_ | -0.04 ± 0.05 | -0.01 ± 0.05 | 0.02 ± 0.05 | 0.04 ± 0.06 |
|  | U_z_ | 0.12 ± 0.04 | 0.12 ± 0.04 | -0.16 ± 0.05 | -0.17 ± 0.05 |
| Body angle (degrees) | χ_GR_ | 18.7 ± 0.8 | 19.3 ± 2.5 | 18.4 ± 0.9 | 19.1 ± 2.6 |
| Average elevation angle (degree) | θ_GR_ | 88.8 ± 2.4 | 88.5 ± 1.9 | 73.4 ± 3.0 | 73.9 ± 2.1 |
| Mid-stroke inclination (degrees) | α | 2.1 ± 2.7 | 3.1 ± 1.6 | 19.3 ± 2.2 | 14.9 ± 1.4 * |
| Stroke plane angle (degrees) | β | 75.3 ± 1.3 | 75.4 ± 2.0 | 71.0 ± 2.6 | 71.9 ± 2.1 |
| Stroke plane amplitude (degrees) | φ_SP_ | 73.4 ± 5.2 | 69.1 ± 3.3 | 72.1 ± 5.8 | 69.2 ± 2.9 * |
| Deviation from stroke plane (degrees) | Θ_SP_ | 14.2 ± 1.4 | 13.1 ± 1.4 | 13.3 ± 1.2 | 13.6 ± 2.9 |
| Frequency | f | 4.97 ± 0.14 | 4.91 ± 1.4 | - | - |
